# A Treatise on the Disease of the Heart and Great Vessels, and on the Affections Which May Be Mistaken for Them

**Published:** 1840-01-01

**Authors:** 


A Treatise on the Diseases of the Heart and Great Vessels, and on the Affections which may be mistaken for them: Comprising the Author's View of the Physiology of the Heart's Action and Sounds, as demonstrated by his Experiments on the Motions and Sounds in 1830, and on the Sounds in 1834-5. By *J. Hope*, M.D. F.R.S. of St. George's Hospital; formerly Senior Physician to the St. Marylebone Infirmary; Extraordinary Member, and formerly President, of the Royal Medical Society of Edinburgh, &c. 8vo. pp. 639. London, John Churchill, 1839.

Dr. Hope informs us, in his Preface to this Edition, that the principal addenda may be found under the following heads:—

1. "The natural sounds of the heart. 2. The sound of costal percussion, with or without tinnitus (Laennec's Cliquetis). 3. Murmurs from valvular disease, and the whole subject of particular valvular diagnosis, which will now, I confidently hope, be found one of the most simple and easy departments of auscultation. 4. Murmurs of the heart and arteries independent of organic disease. 5. Venous murmurs. 6. Musical murmurs. 7. Abdominal murmurs, both connected with pregnancy, and otherwise. 8. Tremor or thrill of the heart, arteries, and veins. 9. Signs, general and physical, of pericarditis and endo-pericarditis. 10. Connection of diseases of the heart with apoplexy, palsy, &c. 11. Partial dilatation or real aneurism of the heart. 12. The signs, physical and general, and the pulse of softening. 13. Signs of adipose disease of the heart. 14. Aneurisms of the aorta bursting into the pulmonary artery and the right ventricle. 15. Abdominal aneurisms. 16. Anæmic, nervous, dyspeptic, plethoric, bilious, and other sympathetic affections of the heart, with their diagnosis. 17. Displacements. 18. The pulses of disease of the heart." vi.

The fiat of public approval may now be admitted to have been conclusively bestowed on this excellent work. Those who were acquainted with Dr. Hope's zeal, labours, and ability, might readily anticipate so much. The work itself has become a standard one, and is probably unequalled in any language, on the subject to which it is devoted. We shall notice, though not at large, some few of the additional facts contained in it, and we trust that our readers will be stimulated to make themselves possessors of the original. We shall turn to the subject of valvular disease, as on that Dr. Hope believes he has thrown much light. He considers, first, the symptoms; secondly, the physical signs.

### 1. *General Symptoms of Disease of the Valves.*

Dr. Hope would assign as general symptoms of disease of the valves, a greatly aggravated form of those which belong to dilatation of the ventricles. These he briefly enumerates as:—cough, copious watery expectoration in many cases, dyspnœa, orthopnœa, frightful dreams and starting from sleep, œdema of the lungs, pulmonary congestion and apoplexy, passive hæmoptysis, (i. e. sputa stained with dark or grumous blood, which occurs especially in great contraction of, or regurgitation through, the mitral valve,) turgescence of the jugular veins, lividity of the face, anasarca and dropsies in general, which in this form of disease attain their utmost degree; injection of any or all the mucous membranes; passive hæmorrhages from the same membranes; engorgement of the liver, spleen, &c. and congestion of the brain, with symptoms of oppression, sometimes amounting to apoplexy; occasionally, cerebral hæmorrhage,—all the worst symptoms, of course, of an advanced case. In the early stages, the hæmorrhages and dropsies are generally absent, and the congestive symptoms less marked. Hæmoptysis is, naturally, more rare in affection of the valves of the right than of the left side. The general symptoms result from retardation of blood in the venous system.

### 2. *Particular Symptoms of Disease of the Valves.*

*a.* When the disease is combined with hypertrophy or dilatation, as is almost invariably the case sooner or later, the symptoms are more severe than those of an equal degree of hypertrophy or of dilatation alone.

*b.* Diseases of certain valves impress, says Dr. Hope, in opposition to the almost universal opinion, well-defined peculiarities upon the pulse. These peculiarities he thus describes.

*The Pulse in Disease of the Mitral Valve.*—When the mitral valve is contracted, and also when it admits of free regurgitation, the pulse is, in various degrees, small, weak, irregular, intermittent and unequal. When either the contraction or the regurgitation is great, the whole of these characters are invariably present. But when the degree of either is slight, (when, for instance, the circumference of the orifice is not diminished more than an inch, or when the aperture for regurgitation is not larger than a goose-quill,) the effect on the pulse may only be a slight degree of weakness and intermittence, increasing when the circulation is hurried.

"Certain other affections, besides disease of the mitral valve, may render the pulse small, weak, intermittent, irregular, and unequal. These exceptions and their diagnosis must, therefore, be briefly noticed. 1. Softening of the heart, as already shown, may occasion all the above characters of the pulse in the highest degree: it may be known by the absence of valvular murmurs. When softening co-exists with mitral disease, the two will, of course, co-operate in producing the pulse in question. 2. The same pulse may be produced by pericarditis with copious effusion compressing the heart, by endocarditis causing polypi in the cavities (Bouillaud,) and by polypus in any other disease of the heart. These diseases may be severally known by their own characteristic symptoms, and by the sudden supervention of the state of pulse. 3. Dyspepsia, nervousness, biliousness, and gout may respectively occasion several, or all of the above qualities of the pulse: they may be known by the attacks of irregularity being only occasional and temporary, and by the absence of valvular murmurs." 378.

*The Pulse in Contraction of the Aortic Valves.*—" Contraction of the aortic valves must be very great to render the pulse small, weak, intermittent and irregular. I have never seen it possess these characters in any marked degree, unless the valves were either soldered together by cartilaginous degeneration, or more or less fixed by ossification in the closed position, so that the aperture was only a limited chink. An induration of the size of an ordinary pea has little effect on the fulness, firmness, and regularity of the pulse, and slighter degrees of contraction appear to have no effect on it whatever." 378.

*The Pulse in Regurgitation through the Aortic Valves.*—Such regurgitation, including that out of the aorta into the right ventricle, or into the pulmonary artery produces a pre-eminently *jerking* pulse, a high degree of the pulse of unfilled arteries, as seen in anæmia from any cause. The diastole or beat of the artery is short and quick, as if the blood were smartly jerked or shot under the finger, the vessel during the intervals feeling unusually empty. This is the most remarkable, appreciable, and constant pulse produced by disease of the heart. In the immense majority of cases, the practitioner may conjecture the disease by this sign alone. It differs from the jerking pulse of anæmia, in being more marked, and in not necessarily being frequent, as the anæmic pulse is, when its jerk is distinct. It may be absent, or scarcely appreciable, if the regurgitation be very slight; and it may be neutralized by free mitral regurgitation or great contraction, in consequence of the enfeebling effects of these lesions on the pulse.

*Valvular Disease of the Right Side* produces, for obvious reasons, little effect on the pulse.

c. " Pain in the region of the heart is another symptom that affords presumptions of disease of the valves. It is true that palpitation or engorgement of the heart may occasion pain, though there be no disease of the valves: I have frequently met with it from these causes in hypertrophy and dilatation. It is likewise true that palpitation may occasion pain, though there be no disease of the heart whatever; I have found it in a large proportion of hysterical, anæmic females and nervous males. But it is when the valves, the coronary arteries, or the commencement of the aorta, are indurated and inelastic, that pain occurs most frequently and with the greatest severity. Sometimes it is little more than an indescribable sense of obstruction or oppression in the præcordial region; but, in other cases, it is an intense lancinating or tearing pain, felt across the præcordia or scrobiculus cordis, (where it might be mistaken for inflammation of the stomach,) and occasionally extending, with a sense of numbness, down the inside of the left arm to the elbow, and sometimes to the fingers. Pain of this description has acquired the name of *angina pectoris.*" 381.

Of the progress, terminations, and prognosis of valvular disease, it is, unfortunately needless to speak. They are too familiar. We therefore pass on to the—

*Physical Signs.*—Dr. Hope informs us that the accession of auscultation to the other means of diagnosis, has rendered it possible to distinguish valvular disease, both in general and particular, with almost complete certainty.

*Signs of Disease of the Aortic Valves.*—One of the various murmurs, *bellows, filing, sawing, rasping,* and *musical,* or *whistling* is heard, writes Dr. Hope, during the ventricular contraction, (i. e, with the first sound,) on the sternum, opposite to the lower margin of the third rib, and thence for about two inches or more upwards, along the course of the ascending aorta towards the right; and it is louder in these situations than below the level of the valves. Its pitch or key is usually that of a whispered *r*, from being superficial, and it accordingly conveys the idea of being pretty near to the ear. When a murmur of this kind is considerably louder along the tract of the ascending aorta than opposite to its valves, and is, at the same time, unusually near-sounding and superficial—in other words, on a higher key than a whispered *r*, it proceeds from disease of the ascending aorta itself. As the murmur from this cause is audible in the situation of the valves, it might lead to the supposition that they also were diseased, and it is sometimes very difficult to ascertain positively that they are not. That a murmur is seated in the aorta, and not in the pulmonary artery, may be known by its being inaudible or very indistinct high up the course of the pulmonary artery, while it is distinct high up that of the aorta. That a murmur is seated in the aorta or its valves, and not in the auricular valves, may be known by its sounding loud and *near* above the aortic valves, where an auricular murmur, if audible at all, sounds feeble, *remote,* and on a low key, like a whispered *who.*

" When," he continues, " there is regurgitation through the permanently open aortic valves, a murmur accompanies the second sound, and its source may be known by the following circumstances :—1. It is louder and more superficial opposite to and above the aortic valves than about the apex of the heart, by which it is distinguished from a murmur in the auricular valves with the second sound. It is louder along the course of the ascending aorta than along that of the pulmonary artery, and down the tract of the left ventricle than down that of the right; by which circumstances its seat is known to be in the aortic, and not in the pulmonic valves. This inference is strongly corroborated by the state of the pulse, which, when the aortic regurgitation is at all considerable, is singularly and pre-eminently jerking—the pulse of unfilled arteries. 3. It is distinguished from a systolic murmur in the aortic orifice by it accompanying the second sound ; by its being more audible, (though with a gradual diminution,) down the course of the ventricle, than a systolic murmur ; by its being prolonged through the whole interval of repose, and even through accidental intermissions of the ventricular contraction (case of W. Esq.) ; and by the weakness of the refluent current always imparting to it the softness of the bellows-murmur, an inferior degree of loudness, and a lower key, like whispering the word *awe* during inspiration. It often becomes musical.

Purring tremor, though necessarily produced by any considerable, salient, or rugged contraction of the aortic valves, can rarely be felt, because the sternum is interposed ; but when the heart is displaced from beneath the sternum, as by hydrothorax, empyæma, emphysema, tumours, consolidation and contraction of one lung and hypertrophy of the other (case of James), &c. the tremor may then become perceptible (case of Mitchell). I have never known it accompany aortic regurgitation." 385.

*Signs of Disease of the Pulmonic Valves.*—"The signs of contraction of the pulmonic valves are the same as those of the aortic, with this difference; that, from the vessel being nearer the surface, the murmur with the first sound seems *closer* to the ear, and is on a higher key, ranging from the sound of a whispered *r* towards that of *s*. I have, however, known it fall below *r* when the circulation was feeble and slow, and the obstruction slight. It may be known that the murmur is not seated in the aorta, by its being inaudible, or comparatively feeble, two inches up that vessel; whereas, at a corresponding height up the pulmonary artery, it is distinct: also, by its being louder down the tract of the right ventricle than down that of the left. It may be known that the murmur does not proceed from regurgitation through the auricular valves, by its being distinct along the course of the pulmonary artery, where auricular murmurs are either wholly inaudible, or very feeble and remote.

When a murmur in the pulmonary artery is considerably louder between the second and third left ribs, close to the sternum, than opposite to the valves, and is there attended with impulse and purring tremor, dilatation of the pulmonary artery may be suspected. In one instance I have known a murmur to be produced by complete ossification of the pulmonary artery penetrating deeply into the lungs.

When there is regurgitation through the pulmonic valves, a murmur accompanies the second sound. Its nature and diagnosis are the same, (the necessary inversions being made,) as in the case of aortic regurgitation, except that the pulse is not jerking." 386.

Disease of the pulmonic valves is so rare that it should not be suspected unless the signs are perfectly marked, or some communication exists between the two sides of the heart.

*Signs of Disease of the Mitral Valve.*—"When the valve is permanently open, admitting of regurgitation, the first sound is attended with a murmur. It may be rough, (rasping,) or smooth, (bellows-murmur,) according to the nature of the contraction, &c. Its key is low,—more or less like whispering *who*; yet it sounds loud and *near* if explored about the apex of the heart, and a little to the sternal side of the nipple. It may thus be easily distinguished from a direct semilunar murmur, which, in this low situation, always sounds feeble and *distant*. The murmur in some cases completely drowns the natural first sound on the left side: in others, the sound can be distinguished at the commencent of the murmur." 387.

Dr. Hope has found perceptible purring tremor produced more frequently by regurgitation through the mitral valve than by any other valvular lesion, especially when the ventricle was hypertrophied and dilated.

"When the mitral valve is considerably contracted, a murmur (best heard in the same situation as the murmur from regurgitation, and distinguishable in the same way from semilunar murmurs,) attends the ventricular diastole and second sound. From the weakness, however, of the diastolic current out of the auricle, the murmur is always very feeble, soft like the bellows-sound, and usually on a rather lower key than a whispered *who*. I have found this murmur absent unless the contraction of the valve was considerable; for the blood had still sufficient room to pass with tranquillity; and I have also found it absent when the contraction was *great*—when, for instance, the aperture admitted one finger only, or merely a quill, provided the current was preternaturally weakened by softening, by extreme dilatation of the heart, or by both. In such cases, however, the mitral disease would not be overlooked, as there is almost invariably a murmur from regurgitation. On the whole, this murmur is exceedingly rare." 388.

*Signs of Disease of the Tricuspid Valve.*—They are the same as those of the mitral, except that the murmurs are loudest on or near the sternum, at the same level as in the case of the mitral—namely, about or a little above where the apex beats. But as the tricuspid valve is rarely so much diseased as to cause a murmur, the practitioner must be very cautious how he pronounces it affected.

*Signs of Disease of the Arterial and Auricular Valves conjointly.*—The murmurs, says our able author, above described as characteristic of each, exist simultaneously in both. The auscultator has merely to take especial care that he explores the arterial murmurs as high up the vessels, and the auricular murmurs as low down the heart as possible. He will thus readily satisfy himself that there are two distinct sources of murmur. It is still easier to determine this, if the murmur attending either sound be of a different *species* in the two situations—if, for instance, the murmur of the aortic or pulmonic valves be of the *soft* bellows-kind, while that of the auricular valve is of the *rough,* grating or rasping kind, or *vice versâ.*

*Diagnosis of Valvular from Inorganic Murmurs.*—Bellows-murmur sometimes exists in the heart, without valvular disease, in anæmic persons, who are generally nervous—in excessive hæmorrhage, and the re-action following it—and in a few cases of hypertrophy with dilatation.

" Murmur from these causes may easily be distinguished from that of valvular disease by the following criteria.

1. It is confined to the aortic orifice, (so far as I have yet discovered,) and to the first sound. Here is one of the great advantages of *particular* valvular diagnosis, as the auscultator can at once exclude the other seven murmurs to which the heart is liable from organic causes only.

2. *It is always weak, and of the soft or bellows kind.*

3. In the anæmic, it is almost invariably attended with a continuous venous murmur in the jugulars, and mostly with a short bellows-whiff, in the carotids, subclavians, and other principal arteries, synchronous with the first sound of the heart.

4. It exists in the anæmic during temporary excitement of the circulation only, subsiding when palpitation ceases and the pulse falls to its natural standard; but as the pulse is permanently quick in considerable anæmia affecting irritable, nervous subjects, especially females; also during the period of reaction after excessive loss of blood, the murmur will persist until the pulse falls by the subsidence of the states in question.

5. The murmur, both of the heart, arteries, and veins, wholly ceases when the anæmia is cured by iron and animal food, the venous murmur being the last that becomes extinct.

When a murmur proceeds from hypertrophy with dilatation, it may be known by its diminishing or ceasing when the action of the heart is calmed, as by repose, venesection, digitalis, &c. In most, if not all cases, this murmur is dependent merely on anæmia, which is very apt to supervene in the advanced stages of hypertrophy with dilatation.

Contrasted with the above, the distinctive characters of valvular murmurs are, 1. That they are not, like inorganic murmurs, restricted to the aortic orifice and first sound, but may be connected with any of the four orifices and with either sound in each: 2. That they persist without intermission for an indefinite length of time, even though the heart be kept in a state of perfect calm: 3.

That they are often of a *rough* character, that is, filing or rasping; whereas, inorganic murmurs have always the softness of the bellows-sound.

Such are the signs which, together with the general symptoms, are, according to my experience, the best for the detection of the diseases of the valves. In the first edition of this work, where the signs were less fully developed, I was enabled to say that 'for several years they had never deceived me as to the general fact whether there was or was not valvular obstruction, and that they had seldom failed to indicate, with perhaps more than necessary precision, the situation of the affection.' I may now venture to add, that, with the improvements introduced in the present edition, the particular diagnosis is even more easy and certain than the general; because a practitioner competent to make the latter only, is more liable to be deceived by inorganic murmurs." 390.

These must be admitted to be very valuable additions to our means of diagnosis in diseases of the heart.

In the following remarks upon the value of an accurate diagnosis, we quite coincide with Dr. Hope. But their application need not be restricted to this particular case. It may be made more wide, and tell as truly of other diseases, as of valvular affections of the heart.

"If it be said that particular valvular diagnosis is a useless refinement, it may be replied that non-auscultators used to say the same of auscultation in general. The truth is, that every improvement in diagnosis is an advantage to the practice of medicine. No one, for instance, will deny the importance of distinguishing inorganic from organic murmurs, as the treatment for the two is diametrically opposite; and this distinction, it has been shown, is remarkably facilitated by particular valvular diagnosis. Again, the pulse, without particular diagnosis, is unintelligible even to the most learned, as Corvisart, Laennec, &c., and has betrayed them into grievous practical errors. Further, disease of certain valves is more injurious and dangerous than that of others. Unless, therefore, the practitioner is able to specify the valve diseased, he cannot nicely adapt his treatment to the exigencies of the case, but must in some instances be uselessly rigid, and in others dangerously lax." 391.

As an appendix, Dr. H. adverts to a few unusual and curious sources of murmur, independent of valvular disease, which might be sources of fallacy.

"1. I had a patient in the St. Mary-le-bone Infirmary, in whom I, as well as the apothecary Mr. Hutchinson, noticed a distinct murmur along the ascending aorta on some occasions, and not the slightest on others. I was much perplexed, and could not make up my mind as to the existence of valvular or aortic disease. The patient died of phthisis, and on post-mortem examination, it was found that the anterior edge of the left lung, completely indurated by tubercular deposition, pressed so exactly on the ascending aorta as actually to have taken its mould, though without adhering. It was now recollected that the murmur had always been heard when she lay on her back or inclined to the right side, but not when inclined to the left: hence we ascribed it to pressure of the lung on the aorta when the position of the body caused it to gravitate towards the right side.

2. Two students of University College called on me, one with slight hypertrophy with dilatation, and violent palpitation from great nervous excitability,—the pulse. for instance, being 120; the other was exempt from organic disease, but affected with violent nervous palpitation, the pulse here also being 120. Both wore very tight waistcoats, preventing the expansion of the lower ribs. During this state of breathing, with the lungs insufficiently inflated, a slight bellows-murmur with the first sound over the semilunar valves existed in both. It was not, however, *exactly* synchronous with that sound, but began an instant later, as if from a separate cause. In both, the murmur ceased *entirely* when, unbuttoning their waistcoats and waistbands of their trowsers, they breathed with the lungs naturally inflated. By alternating the circumstances, the murmur could be created or removed at pleasure. I presume, therefore, that it proceeded from a cause exterior to the heart; and, as the murmur was an instant later than the first sound, the most probable appears to be, that, in the contracted state of the chest, the violent beats of the heart compressed the lung, and, by suddenly expelling its air, created a murmur.

3. Dr. Elliotson mentions two or three cases somewhat analogous. In one —a case of ascites—a bellows-murmur with the first sound, in the region of the left ventricle, instantly ceased on the removal of the fluid from the abdomen; but when it re-accumulated, the sound again became audible. In another case—a young woman with chronic bronchitis, dyspnœa, livid lips and œdematous legs—no murmur existed while she was erect, but it became audible the moment she lay down (Lum. Lecs. p. 18). Dr. Elliotson conjectures that, in the first case, the elevation of the heart by the abdominal fluid might have tilted the organ to an angle with the commencement of the aorta; and, in the second case, he thinks that the cessation of the murmur when the patient was erect, depended on the ventricle being, by gravitation, drawn down more into a straight line with the aorta, when an easier exit was given to the blood. Another conjecture in which he indulges, but to which I cannot assent, is, that dilatation of the right auricle, by pressing against the aorta, might have occasioned the murmur in both.

I have not data by which to decide these points, but the practical inference is, that, in cases of slight bellows-murmur with the first sound, and connected with the arterial orifices, (for the fallacy cannot apply under any other circumstances,) we should not decide till we have ascertained that the murmur continues in the erect as well as the recumbent position, and also while the chest is totally unrestrained by ligatures. I have at present a case of an exceedingly anæmic girl, æt. 17, in whom a venous murmur in the vena innominata was propagated down the great vessels, especially the pulmonary artery, and led a young auscultator into the error of supposing that there was disease of the pulmonic valves." 393.

We have presented the whole, or almost the whole, of Dr. Hope's observations on the diagnosis of valvular diseases of the heart. They tend to throw an unexpected degree of light on affections which before were most obscure, and indeed were given up as enfans perdus in diagnosis.

If we do not at present notice other portions of the volume, it is because the present is not quite a convenient opportunity to do so. We shall couple Dr. Hope's with some other work, and extract such facts or such observations (and they are many) as elucidate a most important, and often an exceedingly obscure class of diseases. In the mean time we recommend this edition very strongly to our readers.

